# Comparison of the efficacy of placental extract gel vs. curcumin gel in the treatment of oral aphthous ulcer: a randomized clinical trial

**DOI:** 10.3389/fdmed.2026.1783623

**Published:** 2026-08-17

**Authors:** Seemadevi Thangeswaran, Naganandini S, Alexander Maniangat Luke, Mohamed Saleh Hamad Ingafou

**Affiliations:** 1 Department of Public Health Dentistry, NIMS Dental College & Hospital, Rajasthan, India; 2 Department of Clinical Sciences, College of Dentistry, Ajman University, Ajman, United Arab Emirates; 3 Center for Medical and Bio-Allied Health Sciences Research (CMBAHSR), Ajman University, Ajman, United Arab Emirates

**Keywords:** canker sores, curcumin, oral mucosal lesions, placental extract gel, recurrent aphthous stomatitis

## Abstract

**Context:**

Canker sores are among the most common oral mucosal lesions with an unclear etiology. Recurrent aphthous stomatitis (RAS) causes considerable pain and discomfort, affecting quality of life.

**Aim:**

To compare the efficacy of placental extract gel (PEG) and curcumin gel in the management of recurrent aphthous stomatitis.

**Settings and design:**

A randomized clinical trial conducted in the Department of Public Health Dentistry.

**Methods and material:**

Thirty participants diagnosed with RAS were randomly allocated into two groups: Group I (PEG, *n* = 15) and Group II (Curcumin gel, *n* = 15). Ulcer size and pain intensity were evaluated at baseline, day 3, and day 7 using standardized clinical measurements and a visual analog scale. Recurrence rates were assessed at 1, 2, 3, and 6 months. Acceptability of the intervention was evaluated using a structured questionnaire.

**Statistical analysis:**

An independent *t-*test, a Chi-square test, and repeated measures ANOVA were used for the analysis, with significance set at *p* ≤ 0.05.

**Results:**

Both groups showed significant reductions in ulcer size and pain scores by day 3 and day 7 (*p* < 0.05), with improvements observed in both interventions. However, the recurrence rate was higher in the curcumin group than in the other group. No adverse effects were reported in either group, although 40% of participants in the curcumin group reported an unpleasant taste.

**Conclusion:**

Both placental extract gel and curcumin gel were effective in reducing pain and ulcer size in recurrent aphthous stomatitis. Placental extract gel demonstrated lower recurrence rates compared with curcumin gel. No adverse effects were observed in either group; however, this study was not powered to detect differences in safety outcomes.

**Clinical Trial Registration:**

https://trialsearch.who.int/Trial2.aspx?TrialID=CTRI/2023/11/059637, Identifier: CTRI/2023/11/059637.

## Introduction

Canker sores, also referred to as oral aphthous ulcer, impact approximately 20%–25% of people and are among the foremost widespread oral mucosal lesions found in the general public (1). It is characterized by solitary or multiple, recurrent and small ulcers with erythematous haloes and yellow/gray floors (2). These ulcers can recur at any stage of life, with recurrence intervals ranging up to 3 months, and their recurrence rate can reach as high as 50% (1). The exact etiology of this disease is unknown, but several contributing elements have been suggested as probable causes, such as hereditary predisposition, localized injury, bacterial components such as *Streptococcus*, *Helicobacter pylori*, and *Lactobacillus*, stress, vitamin and micronutrient deficiencies, underlying systemic diseases such as inflammatory bowel disease (e.g., celiac disease), and endocrine factors such as menopause and pregnancy. Certain drugs such as non-steroidal anti-inflammatory drugs and *ß*-blockers also trigger the development of recurrent aphthous stomatitis (RAS) (3).

Based on size, the frequency and duration of ulcers were classified into RAS minor, RAS major, and herpetiform, with minor RAS being the utmost frequent, accounting for 75%–85% of cases (4). Because of the uncertain etiology of RAS and the unpredictable course of the disease, the fundamental goal of therapy was to control pain or discomfort, foster healing, and prevent recurrence. Several treatment approaches have been studied, including the use of dietary supplements, topical treatments, antibiotics, immunomodulators, corticosteroids, and other pharmacological compounds; however, if these are used for a long duration, various adverse effects may be caused (5). To prevent these adverse effects, phytotherapy as a substitute treatment for RAS have been broadly adopted in several countries over the years primarily because of its relatively minimal chance of causing harmful side effects.

Among all herbal medicines, curcumin is one of the most effective herbals and clinically proved herbal remedy for RAS (5). A systematic review conducted to evaluate the efficacy of curcumin in aphthous stomatitis showed better efficacy than corticosteroids and other remedies (6).

Turmeric, a perennial plant from the ginger family, has been utilized in traditional Indian healing practices for several centuries because of its ability to reduce inflammation, act as an antiallergic, and its antioxidant, antibacterial, and immunomodulatory properties. The plant contains several key active compounds comprising three essential curcuminoids: curcumin (the principal compound, which gives the yellow hue and possesses anti-inflammatory qualities) dimethoxy curcumin, and bisdemethoxycurcumin (7). Curcumin reduces the size of an ulcer, produces less adverse effects, and improves wound healing in RAS. Common standard drugs such as Amlexanox and topical corticosteroids that are used in the treatment of RAS have more adverse effects than other medications, such as secondary candidosis in predisposed patients with salivary hypofunction, prosthesis wearers, users of inhaled corticosteroids, and individuals taking antibiotics (8); also, these drugs are contraindicated in pregnant, lactating mothers, children, and immunocompromised individuals. Hence, curcumin serves as a better alternative medicine for treating RAS (6, 9).

For centuries, the placenta has functioned as a pharmacological agent offering various therapeutic benefits. In numerous countries, the topical application of its extract has been a traditional practice for treating and healing burn injuries, chronic wounds, and postsurgical wounds (10). The placental extract shares similarities with Type III collagen-binding glycoprotein and plays a key role in preserving cell morphology, aiding cell migration, maintaining homeostasis, and promoting the wound-healing process (11).

Recurrent aphthous stomatitis is characterized by painful ulcerations that require therapies capable of reducing inflammation, promoting healing, and preventing recurrence. Curcumin, a bioactive compound derived from *Curcuma longa*, has demonstrated anti-inflammatory, antioxidant, and immunomodulatory properties and has been widely investigated as a herbal therapeutic agent in the management of aphthous ulcers. In contrast, placental extract contains several bioactive components, including growth factors, nucleic acids, glycoproteins, and peptides, which promote tissue regeneration, enhance epithelial cell proliferation, and accelerate wound healing. Although curcumin primarily acts through anti-inflammatory pathways, placental extract may provide additional regenerative benefits by stimulating cellular repair mechanisms. Although both curcumin and placental extract have individually demonstrated beneficial effects in the management of recurrent aphthous stomatitis, direct comparative clinical evidence between these interventions remains scarce. Therefore, the present randomized clinical trial aimed to compare the efficacy of placental extract gel (PEG) and curcumin gel in the management of recurrent aphthous stomatitis and to evaluate recurrence rates following treatment. The null hypothesis is stated as there is expected to be no differences between placental extract gel and curcumin gel in terms of clinical improvement in patients with recurrent aphthous ulcer.

## Materials and methods

Our randomized clinical trial was planned and performed at (23) the Department of Public Health Dentistry, NIMS Dental College and Hospital, Rajasthan, India. Our study was validated by the Institutional Ethical Committee Board (IEC of NIMS University, Rajasthan NIMSUR/IEC/2023/566), and the trial was registered in the CTRI registry with Ref no. CTRI/2023/11/059637 (Registered on 7 November 2023). The procedures conformed to the ethical standards set by the relevant committee on human experimentation (institutional or regional) and adhered to the Helsinki Declaration of 1975, updated in 2024.

The sample size was calculated using G*Power software (23) (version 3.1) based on the primary outcome variable of ulcer size reduction between Group I and Group II patients. The calculation was performed using a two-tailed test with a significance level (*α*) of 0.05 and statistical power of 80% (*β* = 0.20).The sample size estimation was based on a between-group comparison, with an effect size (Cohen's *d* = 0.71) derived from previously published studies that evaluated similar interventions (7, 11). In these studies, the reported mean differences (approximately 2–5 mm^2^) and standard deviations (approximately 3–6 mm^2^) corresponded to an effect size. Based on these parameters, in this study, the minimum required sample size was estimated to be 26 participants. To compensate for potential dropouts and ensure adequate statistical power, the final sample size was increased to 30 participants, with 15 participants allocated to each group.

The study's allocation sequence was generated using the Randomization Main software (12). During the registration process, each patient was assigned a unique identification code. A software-generated table containing 30 arbitrary numbers was split into two separate groups and assigned to the two groups followed by a 1-to-1 assignment ratio.

A separate researcher responsible for maintaining the code ensured that the intervention strategy remained blinded by securing it in sealed envelopes labeled with sequential numbers. This approach ensured that neither the principal investigator nor the participants got to know about the assignments. The 30 patients were then randomly allocated into two groups using a computerized random number method.

Group I: 15 patients (placental extract gel).

Group II: 15 patients (Curcumin gel).

All gels used in this study were identically packaged, computer-tagged, and allocated by a second investigator who held the key to the treatment assignments. Following the baseline parameter measurements by the principal investigator, participants were randomly allocated coded tubes through a computer program guaranteeing impartiality regardless of age or gender. The randomization process was carried out by a separate researcher who was unaware of the study parameters. The study followed a double-blind design, where both the participants and the investigator responsible for clinical assessment were unaware of the treatment allocation. The gels used in both groups were identically packaged and contained coded tubes to maintain blinding. A second investigator, who was not involved in patient assessment or data analysis, was responsible for labeling and distributing the coded gels. The treatment codes were securely maintained and were revealed only after completion of the study and final data analysis. A statistical analysis was performed after decoding the intervention groups to ensure an unbiased analysis.

### Inclusion criteria

The inclusion criteria for the study were as follows: individuals of either gender and 18 years and above who had been diagnosed with recurrent aphthous ulcer. Patients presenting with ulcers of less than 48 h duration were included to ensure that the lesions were assessed during the early stage of ulcer development before substantial spontaneous healing occurred. Early-stage ulcers provide more standardized baseline measurements and allow accurate evaluation of treatment effects. Similar inclusion criteria have been used in previous clinical trials that evaluated therapeutic interventions for recurrent aphthous stomatitis. In addition, individuals who experienced recurrent aphthous ulcers with at least two previous episodes and who were willing to be treated for aphthous ulcer were considered for scheduled follow-ups.

### Exclusion criteria

The exclusion criteria were as follows: patients suffering from any systemic disorders and allergic to drug material, pregnant woman and lactating mothers, and subjects with alcohol and tobacco habits. Patients who were on topical corticosteroids, NSAIDs, systemic antibiotics, immune-modulatory agents, or other intraoral topical medications were excluded from the study.

### Intervention

In this study, Placentrex® gel (Albert David Ltd., India) containing placental extract and Curenext® oral gel (Abbott Healthcare Pvt. Ltd., India) containing *Curcuma longa* extract were used as the intervention and comparator, respectively. Placentrex® gel contains 0.1 g extract of fresh human placenta per gram of gel, with total nitrogen not exceeding 0.25% w/w. Curenext® oral gel contains *Curcuma longa* (rhizome) extract 10 mg per gram, along with a gel base and excipients, including brilliant blue FCF (For Coloring Food), erythrosine, and titanium dioxide.

### Treatment groups

Both formulations exhibit good mucosal adherence and are designed to remain at the site of application without dissolving rapidly in saliva, allowing prolonged therapeutic action. Participants were instructed to gently dry the ulcer area using sterile cotton or a clean cloth prior to application to enhance adherence and absorption of the gel. The study participants in both groups were instructed to apply the assigned gel directly over the ulcer using their fingertips, ensuring that the lesion was completely covered. They were asked to apply the gel three times daily (after each meal and before bedtime) for a duration of 7 days. The participants were also advised to avoid consuming food or fluids for 1 h following gel application to allow adequate mucosal contact time and optimal therapeutic effect. All patients were required to continue the prescribed therapy daily until the treatment was complete and were strictly instructed to refrain from using any other topical or systemic medications such as steroids, non-steroidal drugs, or antihistamines from the onset of the study (day 0) until its completion (day 7) to avoid any interference with the results.

### Clinical assessment

The diagnosis of recurrent aphthous stomatitis was primarily based on clinical examination and patient history. A cytological smear examination was performed only in selected patients who presented with atypical features, such as unusually large ulcers, prolonged duration, irregular margins, or non-healing lesions, to exclude other ulcerative or infectious conditions. Smears were obtained from the ulcer base using a sterile swab, which was air-dried and examined under light microscopy to identify inflammatory cellular features consistent with aphthous ulceration.

The participants in both interventions were examined based on progress in pain intensity, changes occurring in the ulcer area, and decrease in erythema, and a psychological assessment was also done using DASS-21 (Depression Anxiety Stress Scale-21) (13, 24). The data acquisition process was initiated at baseline, i.e., day 0, day 3, and day 7, in the Department of Public Health Dentistry.

Pain evaluation was conducted using a 10 cm visual analog scale (VAS). This scale is a horizontal line with endpoints indicating no pain and the worst possible pain. This scale was used to evaluate unprovoked pain (pain experienced spontaneously without any external irritation to the ulcer). The participants were asked to mark the line at the point with the highest precision to reflect their present state of ulcer pain.

The types of aphthous ulcer, the location/site of lesions, and the number of ulcers at the day of the examination were also recorded. Ulcer size was assessed using a standardized William's periodontal probe. It has mm gradations for precise measurement. Two measurements were taken: The first measurement represented the ulcer's longest diameter, while the second was taken at a right angle to the first. The ulcer's surface area was measured in mm^2^ and calculated by multiplying the two measurements. This method provides a simple and reproducible clinical approximation of ulcer size and has been commonly used in previous clinical studies that evaluated treatments for recurrent aphthous stomatitis (11).

The recurrence rate of the ulcer was monitored during the 1st, 2nd, 3rd, and 6th months. The study participants were asked to maintain a diary for 6 months to record the presence of ulcer. At the end of 6 months, we contacted the participants over the phone and asked them to send the details of recurrence.

All clinical assessments were done by a single investigator who was calibrated before performing the examinations.

To assess the level of patient acceptance of the intervention, the study participants were prompted to complete the questionnaire. The questionnaire guide included concerns about the color, odor, and taste of the gel as well as any adverse effects due to the intervention. The questionnaire was filled by the participants on the 7th day when they turned up for follow-up.

### Statistical analysis

The collected data were analyzed and tabulated using various statistical tests. A normality test (Kolmogorov–Smirnov) was conducted to assess the normal distribution of the samples. Descriptive statistics were presented as mean ± standard deviation (SD). An independent sample *t*-test was employed to compare the differences between the two groups. For categorical variables, a Chi-square test was used, and repeated measures of ANOVA were employed for comparisons within groups. Statistical significance was set at a *p*-value of ≤0.05. The primary outcome of the study was ulcer size reduction, and secondary outcomes were pain reduction (VAS) and recurrence rates. Analyses of secondary outcomes were considered exploratory in nature. The primary analysis of this study was based on unadjusted comparisons of outcomes between the two groups using repeated measures ANOVA and independent sample *t*-tests. In addition, an analysis of covariance (ANCOVA) was performed to adjust for baseline ulcer size as a covariate, to provide a more robust assessment of treatment effects. Statistical analyses were conducted using SPSS software for Windows, version 24.0.

## Results

**Figure 1:**
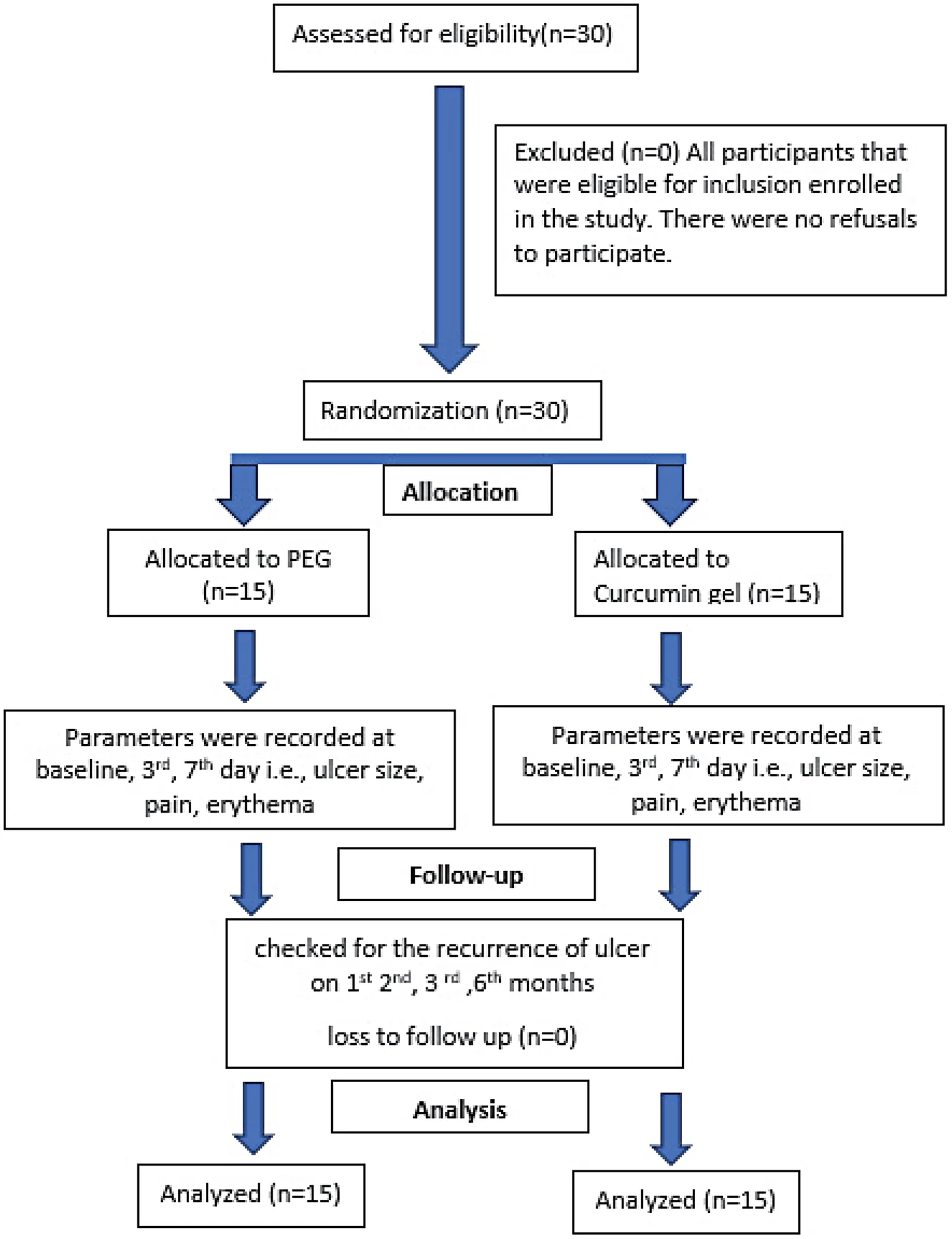
A consort flow diagram.

The study comprised 30 participants equally distributed between the two genders, females (*n* = 15) and males (*n* = 15). The participants had an average age of 24 years, with their ages spanning from 19 to 30 years. Males [*n* = 9 (30%)] were predominantly included in Group I (PEG), while females [*n* = 11 (36.7%)] were more in number in Group II (curcumin). No notable statistical difference (*p* > 0.05) was detected between the PEG and curcumin gel groups with regard to demographic data, i.e., age and gender. A participant flow diagram is presented and described in Figure 1. The lesions were most commonly present in the labial mucosa 9 (30%), followed by the lateral border of the tongue 8 (26.6%), lip 6 (20%), buccal mucosa 5 (16.6%), and soft palate 2 (6.6%).

Baseline psychological variables such as stress, anxiety, and depression were assessed in both groups prior to the intervention. The distribution of stress, anxiety, and depression levels between the placental extract gel group and the curcumin gel group showed no statistically significant difference (*p* > 0.05). This indicates that both groups were comparable in terms of psychological status at baseline. The DASS is presented in Table 1.

**Table 1:** Distribution of the levels of stress, anxiety, and depression between the two groups.

Psychological variables		Group I (PEG), *n* (%)	Group II (curcumin), *n* (%)	*p*-Value
Stress	Absent	2 (13.3)	3 (20)	0.4
	Mild	7 (46.6)	6 (40)	
	Moderate	4 (26.6)	3 (20)	
	Severe	2 (13.3)	3 (20)	
Anxiety	Absent	3 (20)	4 (26.6)	0.067
	Mild	6 (40)	6 (40)	
	Moderate	5 (33.3)	3 (20)	
	Severe	1 (6.6)	2 (13.3)	
Depression	Absent	4 (26.6)	5 (33.3)	0.56
	Mild	5 (33.3)	4 (26.6)	
	Moderate	3 (20)	4 (26.6)	
	Severe	3(20)	2(13.3)	

PEG, placenta extract gel.

**Figure 2:**
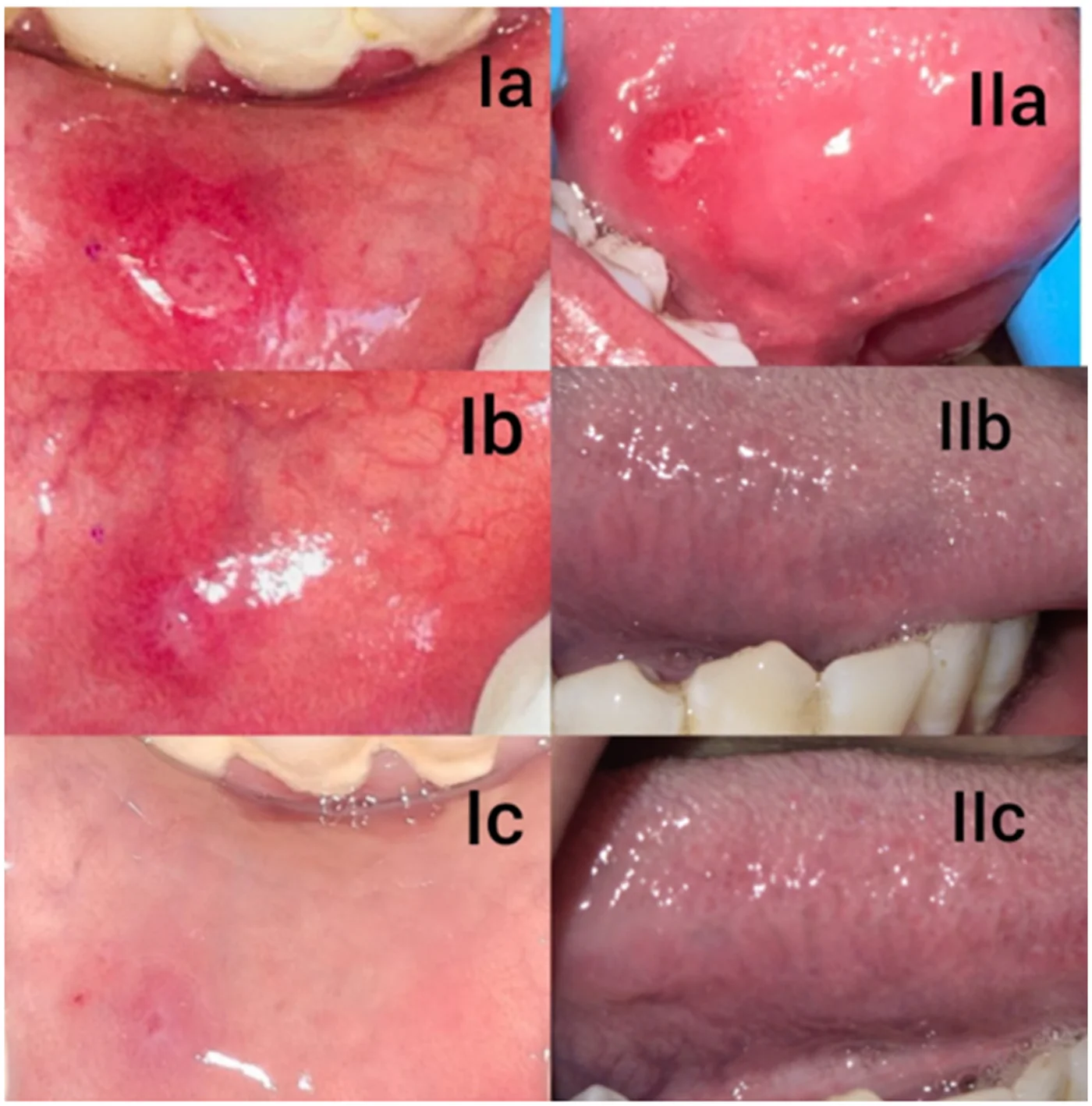
Image I depicts ulcer treated with the placental extract gel: **(la)** ulcer at baseline, **(lb)** ulcer at the 3rd day, and **(Ic)** ulcer at the 7 days. Image II depicts ulcer treated with curcumin: **(Ila)** ulcer at baseline, **(llb)** ulcer at the 3rd day, and **(llc)** ulcer at the 7th day.

**Figure 3:**
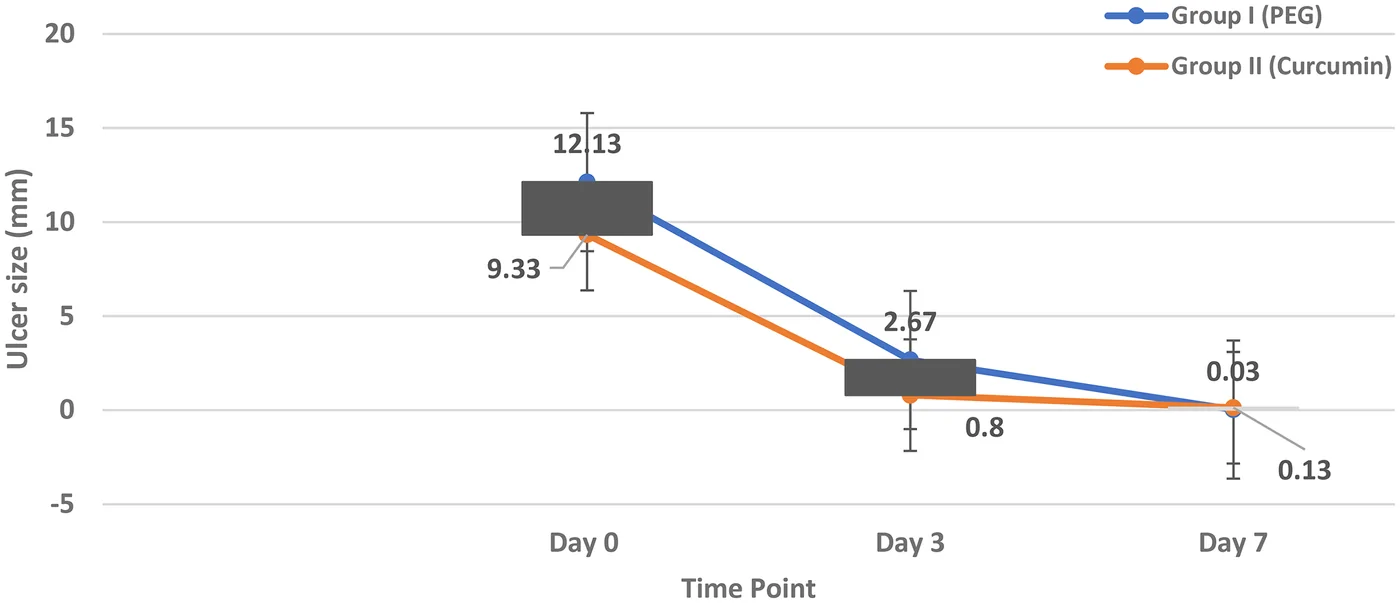
Ulcer size between the two groups.

**Figure 4:**
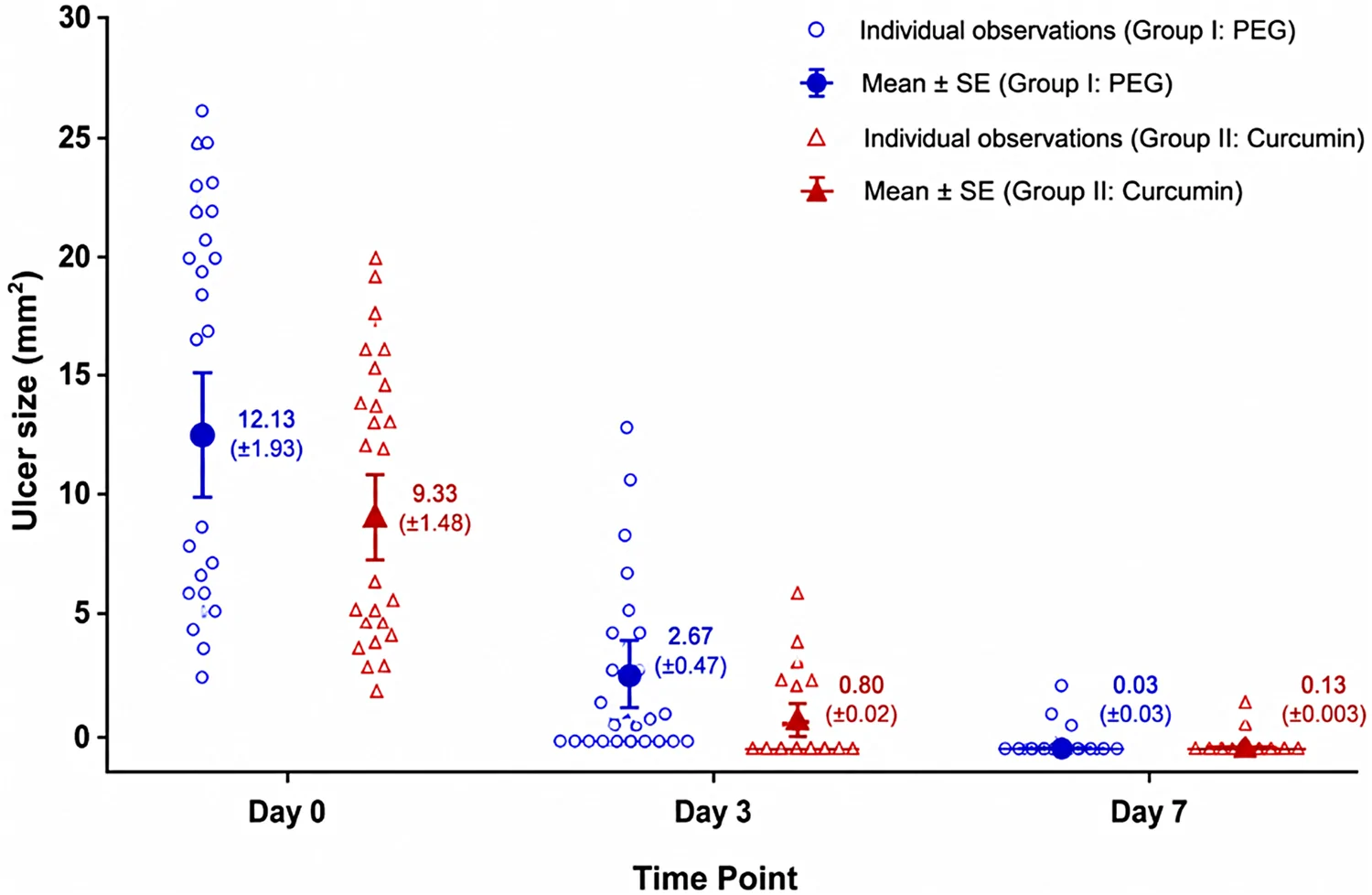
Ulcer size changes over time: jittered individual observations with mean ± SE.

With regard to ulcer size, the placental extract gel group showed a decrease in mean ulcer size (mm^2^) from baseline (12.13 ± 7.46) to day 7 (0.03 ± 0.1), which was highly significant (*p* < 0.05) at both intervals (day 3 and 7). In the curcumin group, the size of the ulcer was reduced from baseline (9.33 ± 5.71) to day 7 (0.13 ± 0.01), which was also highly significant. The mean difference in ulcer size at day 7 between the two groups was 0.10 mm^2^ (95% CI: 0.04–0.16), indicating a statistically and clinically significant difference favoring the PEG group. Intergroup comparisons of groups also demonstrated a significant difference in the size of ulcer (*p* < 0.05) (Table 2, Figures 2–4). An ANCOVA analysis adjusting for baseline ulcer size showed no statistically significant difference between the groups (*F* = 0.98, *p* = 0.33) (Table 3).

**Table 2:** Comparison of ulcer size at different intervals.

Groups	Day 0	Day 3	Day 7	95% confidence intervals	*p*-Value	Mean difference
Group I (PEG) *n* = 15	12.13 ± 7.46	2.67 ± 1.83	0.03 ± 0.10	−0.02 to 0.08	<0.001^a^*	−0.10
Group II (curcumin) *n* = 15	9.33 ± 5.71	0.80 ± 0.06	0.13 ± 0.01	0.12–0.14		
*p*-Value	0.25^b^	0.02^b^*	0.01^b^*			

a Repeated measures of ANOVA.

b Independent sample *t*-test.

* Significant values.

**Table 3:** ANCOVA for ulcer size at Day 7 adjusted for baseline ulcer size.

Source	Sum of squares	df	Mean square	*F*-value	*p*-Value
Baseline ulcer size (covariate)	2.56	1	2.56	0.51	0.48
Group (PEG vs. curcumin)	4.90	1	4.90	0.98	0.33
Error	135.00	27	5.00		

**Figure 5:**
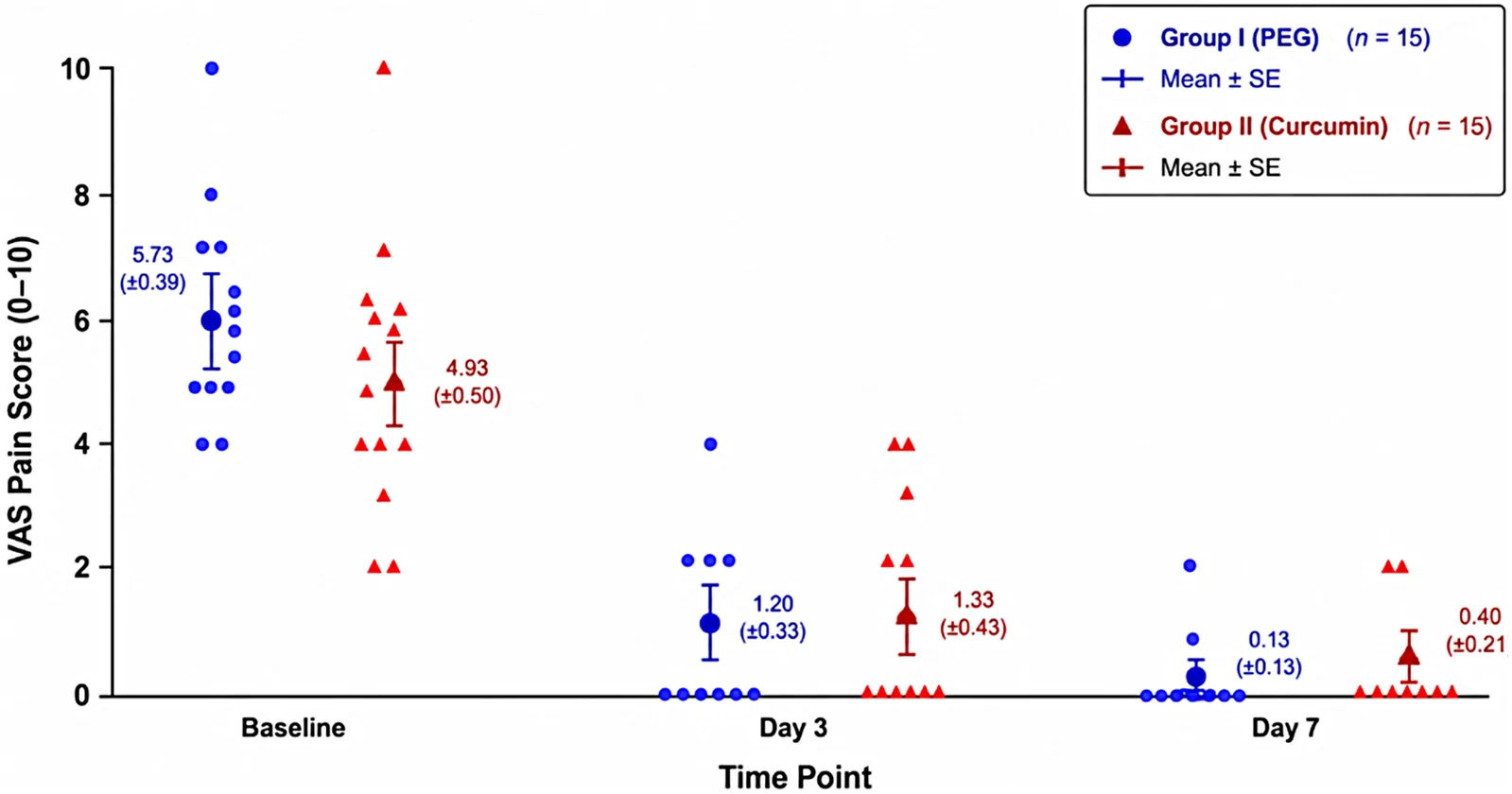
The VAS score changes over time: jittered individual observations with mean ± SE.

The PEG group exhibited a significant reduction in pain scores from baseline (5.73 ± 2.49) to day 7 (0.13 ± 0.51). The curcumin group pain scores also significantly reduced from baseline (4.93 ± 1.83) to day 7 (0.40 ± 0.82). Upon a comparison of the two groups, PEG exhibited a more observed improvement with a statistically meaningful decrease (*p* < 0.05) in VAS at both day 3 and 7 in contrast to the curcumin group. The mean difference in pain scores at day 7 was 0.27 (95% CI: 0.01–0.53), reflecting a moderate but clinically meaningful improvement (Table 4, Figure 5).

**Table 4:** Comparison of pain scores (VAS) at different intervals.

Groups	Day 0	Day 3	Day 7	95% confidence intervals	*p*-Value	Mean difference
Group I (PEG) *n* = 15	5.73 ± 2.49	1.20 ± 1.26	0.13 ± 0.51	0.15–0.41	0.01^a^*	0.27
Group II (curcumin) *n* = 15	4.93 ± 1.83	1.33 ± 1.63	0.40 ± 0.82	0.06–0.86		
*p*-Value	0.23^b^	0.02^b^*	0.03^b^*			

a Repeated measures of ANOVA.

b Independent sample *t*-test.

* Significant values.

**Figure 6:**
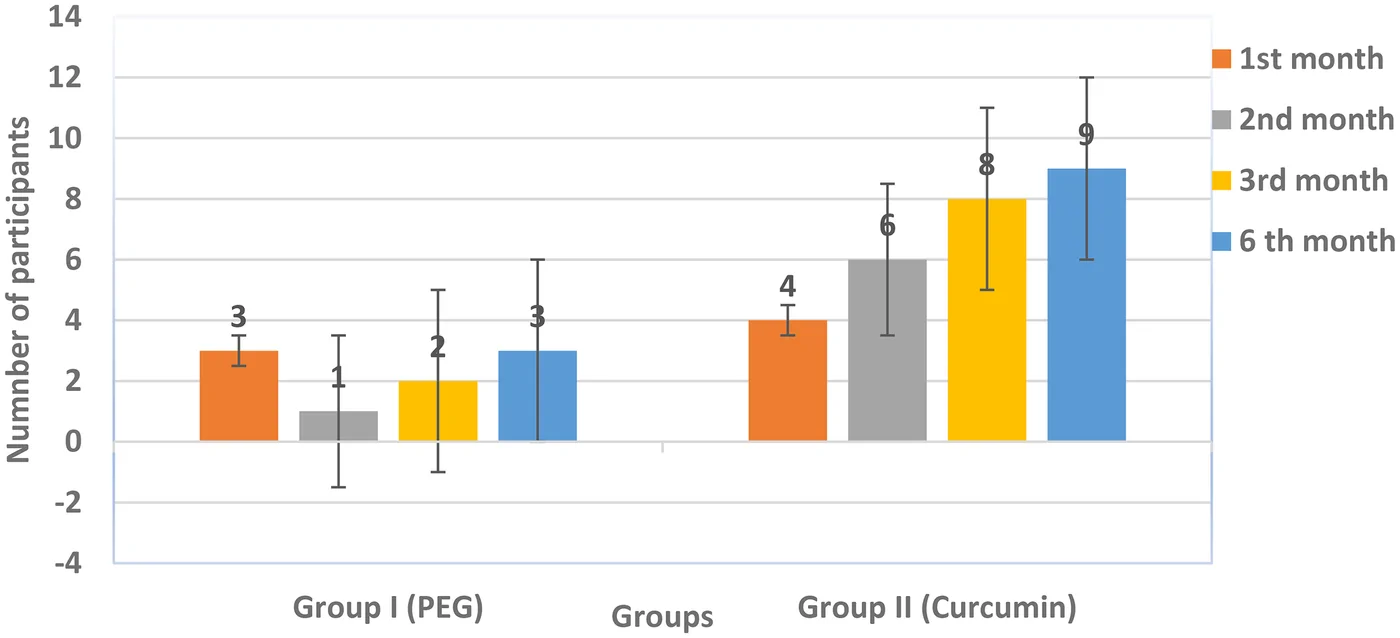
Comparison of recurrence.

The recurrence rate was assessed in both groups at 1, 2, 3, and 6-month intervals. The placental extract gel group showed lower recurrence rates compared with the curcumin group at all time points (Table 5, Figure 6).

**Table 5:** Comparison of recurrence in both groups.

Groups	Recurrence at different time points							
	1st month, *n* (%)	*p*-Value	2nd month, *n* (%)	*p*-Value	3rd month, *n* (%)	*p*-Value	6th month, *n* (%)	*p*-Value
Group I (PEG)	3 (20)	0.5^a^	1 (5)	0.01^a^*	2 (13)	0.05^a^*	3 (20)	0.005^a^*
Group II (curcumin)	4 (27)		6 (40)		8 (53)		9 (60)	

^a^Fisher's exact test.

*Significant values.

Both groups tolerated the medication well with no reported adverse events, hypersensitivity reactions, and complaints about the gels. All participants found that both gels were acceptable. However, the curcumin group (six out of 15) of individuals reported that the taste of the gel was not pleasant.

## Discussion

The management of recurrent aphthous stomatitis (RAS) remains challenging because of its multifactorial etiology and the absence of a definitive treatment modality. Current therapeutic approaches primarily focus on symptom relief, including reduction of pain, acceleration of ulcer healing, and prevention of recurrence (13). To the best of our knowledge, there is limited comparative evidence that evaluates placental extract gel and curcumin gel in the management of RAS.

Conventional treatment modalities such as topical and systemic corticosteroids, cauterization, antibiotics, and other agents mainly provide symptomatic relief (5). In contrast, the placental extract used in the present study is known to promote tissue repair and regeneration. It contains multiple growth factors, including vascular endothelial growth factor, transforming growth factor, fibroblast growth factor, platelet-derived growth factor, and epidermal growth factor, which contribute to enhanced wound healing, modulation of inflammation, and stimulation of cellular proliferation (10, 14). The placental extract gel utilized in this study was reported by the manufacturer to be free from HIV antibodies, HCV antibodies, and hepatitis B surface antigen, thus ensuring its safety for human use.

Curcumin, a bioactive compound derived from *Curcuma longa*, has been widely used in traditional medicine (15). It possesses antioxidant, anti-inflammatory, and immunomodulatory properties (16). Curcumin modulates inflammatory pathways by suppressing proinflammatory cytokines such as tumor necrosis factor-alpha (TNF-α), interleukins (IL-4, IL-6, IL-10), and adhesion molecules, thereby reducing inflammation and promoting ulcer healing (17, 18).

The etiopathogenesis of RAS has been associated with microbial factors and oxidative stress (19–21). Both placental extract and curcumin exhibit have antibacterial and antioxidant properties, which may contribute to their therapeutic effects in enhancing ulcer healing (22).

The findings of the present study demonstrated that both PEG and curcumin gel were effective in reducing ulcer size and pain scores from baseline to day 7. These results are consistent with those of a systematic review by Al-Maweri et al. (9), who reported observed reductions in ulcer size and pain with curcumin therapy. Similarly, the findings align with those of a study by Morsy (11), who observed significant reductions in ulcer size and pain with the placental extract gel.

In our study, the results reflected that both the PEG and curcumin groups demonstrated a substantial decline in ulcer dimensions from baseline to day 3, with minimal differences observed between the two groups (0.13 ± 0.51 vs. 0.40 ± 0.82) on day 3. The mean VAS score decreased in both groups from baseline to day 3, with a more significant reduction observed in the PEG group (0.13 ± 0.51) than in the curcumin group (0.40 ± 0.82). The discrepancy between unadjusted and adjusted analyses observed in this study may be attributed to baseline differences in ulcer size and the relatively small sample size. The ANCOVA model, which accounts for baseline variability, did not demonstrate a statistically significant difference between the two treatment groups. Recurrence rates were monitored in both groups at 1, 2, 3, and 6-month intervals, with both reporting recurrences. However a higher recurrence rate was observed in the curcumin group than in the PEG group at 2, 3,, and 6 months. This difference may be attributed to the greater healing ability of the placental extract gel, which accelerates the healing process and repairs keratinocytes through epidermal growth factors. These factors make the tissues less prone to oxidative stress and more resistant to external stimuli, thereby contributing to the lower recurrence rate in the PEG group.

Both interventions were well tolerated, with no adverse effects reported in either group, indicating a favorable safety profile. However, 40% of participants in the curcumin group reported an unpleasant taste, which may influence patient acceptability and adherence. Modifications in formulation, such as the addition of flavoring agents, may improve palatability and compliance.

Despite these findings, certain limitations in this study should be considered. Recurrence assessment was based on patient-maintained diaries and telephonic follow-up, which may introduce recall bias and reliance on self-reported data. These could potentially lead to overestimation of treatment effects. In addition, compliance with the intervention was assessed through self-reporting, which may not accurately reflect actual adherence.

Furthermore, variations across different populations may influence the generalizability of the findings. Future studies with larger and more diverse populations, as well as placebo-controlled or multiarm trial designs, are recommended to further validate these results and strengthen the evidence regarding the effectiveness of PEG in the management of RAS.

## Conclusion

Within the limitations of the present study, both placental extract gel and curcumin gel demonstrated comparable safety profiles and effectiveness in the management of recurrent aphthous ulcers, with the placental extract gel showing a slightly greater reduction in the recurrence of ulcer.

## Data Availability

The original contributions presented in the study are included in the article/Supplementary Material, further inquiries can be directed to the corresponding author/s.
